# Machine learning-based approach to guide the choice between baricitinib and tocilizumab in critical COVID-19 pneumonia treatment: a retrospective cohort study

**DOI:** 10.3389/fmed.2025.1734109

**Published:** 2026-01-07

**Authors:** Euijin Chang, Myung-Soo Kim, Se Yoon Park, Kyungsup Kwon, Hyeon Mu Jang, So Yun Lim, Seongman Bae, Jiwon Jung, Min Jae Kim, Yong Pil Chong, Sang-Oh Lee, Sang-Ho Choi, Yang Soo Kim, Gyucheol Choi, Sungwon Lim, Jamin Koo, Sung-Han Kim

**Affiliations:** 1 Department of Infectious Diseases, Asan Medical Center, University of Ulsan College of Medicine, Seoul, Republic of Korea; 2 ImpriMedKorea, Inc., Seoul, Republic of Korea; 3 Department of Internal Medicine, Hanyang University College of Medicine, Seoul, Republic of Korea; 4 ImpriMed, Inc., Mountain View, CA, United States; 5 Department of Chemical Engineering, Hongik University, Seoul, Republic of Korea

**Keywords:** coronavirus disease 2019, pneumonia, machine-learning, baricitinib, tocilizumab

## Abstract

**Introduction:**

Clear guidance on choosing baricitinib (BCT) or tocilizumab (TCZ) for critical COVID-19 pneumonia remains limited. We developed machine-learning (ML) models to inform immunomodulator selection.

**Methods:**

We curated clinical data from patients with critical COVID-19 pneumonia admitted between January 2020 and June 2024. Development cohort (*n* = 390) was split 4:1 into training and validation sets, with Day-90 mortality as endpoint. For each therapy, patients were labeled high risk when model-predicted mortality exceeded F1-optimized thresholds. External validation used a test cohort (*n* = 95). A combinatorial risk stratification assigned patients to four groups: I (low risk for both), II (low risk for TCZ, high risk for BCT), III (high risk for TCZ, low risk for BCT), and IV (high risk for both). Survival was compared for TCZ- and BCT-treated patients within each group.

**Results:**

TCZ and BCT models achieved ROC-AUCs of 0.81 and 0.84, with test accuracies of 0.67 and 0.77, respectively. In test cohort, survival differed significantly between high- and low-risk strata for each agent. In Group II, mortality was significantly higher with BCT than TCZ (hazard ratio (HR) 2.32, *p* = 0.032); in Group III, mortality was significantly higher with TCZ than BCT (HR 3.34, *p* < 0.001). Model-guided selection would have changed therapy in 13.2% (65/492) of patients; as the models are prognostic rather than causal, any survival benefit from the alternative agent remains hypothetical.

**Conclusion:**

ML models may support treatment selection between BCT and TCZ in patients with critical COVID-19 pneumonia. Prospective studies are warranted to assess whether model-guided choices improve survival and to validate generalizability across clinical settings.

## 1 Introduction

Coronavirus disease 2019 (COVID-19) remains one of the leading causes of global morbidity and mortality, accounting for an estimated 420 million disability-adjusted life years in 2020-2021 (1). More than seven million deaths were reported worldwide between December 2019 and December 2024 (2). Although disease severity has generally declined with successive variants, COVID-19 continues to cause substantial mortality among the elderly and immunocompromised patients (3, 4). Mortality associated with COVID-19 is primarily attributed to critical COVID-19 pneumonia, which often requires oxygen supplementation through high-flow nasal cannula (HFNC) devices, noninvasive ventilation (NIV), mechanical ventilation (MV), or extracorporeal membrane oxygenation (ECMO) (5). Consequently, effective treatment for critical COVID-19 patients is essential to reduce mortality and improve global health outcomes.

The progression of critical COVID-19 pneumonia is driven by excessive secretion of inflammatory cytokines along with viral replication (6). Immunomodulatory agents such as baricitinib (BCT) and tocilizumab (TCZ), in conjunction with dexamethasone and remdesivir, are recommended for treating critically ill COVID-19 patients (5). Both BCT and TCZ alleviate hyperinflammation processes during COVID-19 pneumonia by inhibiting Janus kinase (JAK) 1/2 and interleukin-6 (IL-6) receptors, which drive and modulate inflammatory signaling pathways (7, 8). Several randomized controlled trials have demonstrated that BCT and TCZ can significantly reduce disease progression and mortality in critically ill COVID-19 patients (9–11). Thus, the combination of immunomodulators such as BCT and TCZ with dexamethasone is considered superior to relying solely on antiviral agents like remdesivir for treating critical COVID-19 pneumonia.

However, no clear guidelines currently exist for selecting between BCT and TCZ when treating critical COVID-19 pneumonia. Although previous studies have compared their efficacy, no significant differences between the two have been reported (12–14). One recent retrospective study found that BCT was associated with lower risks of mortality and intubation after adjustment for confounders, but randomized trials are still needed to confirm these findings (15). Consequently, the selection of immunomodulators often relies on clinicians’ experience, patient comorbidities, and current clinical status. Although this approach is valuable, it remains uncertain whether this method effectively improves survival outcomes in patients with critical COVID-19 pneumonia. Personalized therapeutic approaches are therefore needed to guide the selection of either BCT or TCZ in a manner that optimizes survival.

Machine learning (ML) offers a promising approach for personalizing treatment by analyzing not only physicians’ clinical experience and patient data but also by identifying complex, high-dimensional patterns that may not be immediately apparent to physicians (16). Several studies have demonstrated the utility of ML in developing personalized treatment strategies across various clinical settings (17, 18). Additionally, ML has been employed to accurately diagnose COVID-19 infections and to predict their severity and progression (19, 20). However, few studies have explored the use of ML approaches in guiding the selection of immunomodulators for treating critically ill COVID-19 patients.

This study aims to develop an ML model to assist in selecting between BCT and TCZ in patients with critical COVID-19 pneumonia. By predicting treatment responses and outcomes, this model aims to improve survival outcomes through personalized treatment recommendations. Using the ML-based approach, we estimate survival rates when patients were treated with BCT or TCZ. We also apply ML-based risk stratification to group patients, predict the most appropriate immunomodulator for each group, and assess improvements in survival rates.

## 2 Methods

### 2.1 Patient selection and treatments

This retrospective study included adults aged 18-80 admitted to Asan Medical Center (AMC) in the Republic of Korea from January 2020 to June 2024, with critical COVID-19 pneumonia requiring HFNC, NIV, MV, or ECMO (5). Severe Acute Respiratory Syndrome Coronavirus 2 (SARS-CoV-2) infection was confirmed by nasopharyngeal polymerase chain reaction or rapid antigen testing. Patients received either BCT or TCZ in addition to standard of care (SOC) and were followed for 90 days after initiation of immunomodulators.

During the study period, three variants of concern successively predominated in the Republic of Korea: Alpha (January 2020–July 2021), Delta (August 2021–January 2022), and Omicron (February 2022–June 2024). Treatments were administered in a non-randomized manner and were dependent on guidelines and drug availability (5). SOC for critical COVID-19 pneumonia included appropriate respiratory support based on disease severity, remdesivir, dexamethasone, low-molecular-weight heparin, and immunomodulators such as BCT or TCZ. Remdesivir became available in the Republic of Korea in July 2020 and dexamethasone was introduced into SOC for patients requiring oxygen support in July 2020, following results from a large-scale randomized controlled trial (21).

BCT or TCZ was administered to patients with rapidly deteriorating severe or critical COVID-19 pneumonia. BCT was approved for the treatment of COVID-19 pneumonia in April 2021, while TCZ was approved in March 2022. Immunomodulator choice was individualized based on clinical status and physician preference; TCZ was favored for chronic kidney disease or dysphagia, whereas BCT was preferred when bacterial/fungal coinfection was suspected, given long half-life of TCZ. Contraindications for each agent are listed in Supplementary Table 1 (22, 23).

### 2.2 Development of machine-learning models

Clinical data from the development cohort were extracted from the Asan Biomedical Research Environment (ABLE) system at AMC. A total of 16 underlying diseases and 38 clinical characteristics (Supplementary Methods) obtained around admission and immunomodulator initiation were included as features for the ML models predicting outcomes following BCT or TCZ treatment. The corresponding dataset for the independent test cohort was obtained from Hanyang University Medical Center (HYUMC) in the Republic of Korea. This study was approved by the Institutional Review Boards (IRB) of AMC (IRB No. 2021-0024) and HYUMC (IRB No. 2023-09-037), with an informed consent waived.

The datasets comprised both categorical and numerical variables. Within the development cohort, the dataset, consisting of input variables and treatment outcomes, was randomly split into a training set and an internal validation set in a 4:1 ratio, in a stratified manner with respect to Day-90 mortality. This internal validation set was used for final model performance assessment before external validation. The HYUMC cohort was reserved as an independent external test set. Categorical variables were transformed into numerical values through label encoding, while numerical variables were preprocessed using the MinMax scaler (24). Missing values (proportions described in Supplementary Table 2) were imputed using the median of the non-missing values in the training set for each variable. The approach was chosen after repeating the entire model-development pipeline under alternative imputation strategies—minimum, maximum, kNN, and MICE (25, 26).

The ML models were trained to predict Day-90 mortality under BCT or TCZ treatment. Model development, including feature selection and hyperparameter tuning, was performed exclusively within the primary training set. A minimal feature subset was first identified using permutation feature importance with an artificial neural network (27, 28). Subsequently, to robustly evaluate candidate feature sets and optimize model hyperparameters, we employed recursive feature elimination. This process utilized repeated stratified 5-fold cross-validation (100 resampling iterations per combination of features/hyperparameters), and the combination yielding the highest mean F1-score across the cross-validation folds was selected (29). Final models were then refitted on the entire training set using the selected features and hyperparameters and subsequently evaluated on the previously unseen HYUMC test cohort.

### 2.3 Treatment-specific risk stratification based on machine-learning models

Machine-learning models were employed to stratify patients into treatment-specific risk groups. TCZ-treated patients were stratified by predicted Day-90 mortality using both the TCZ-specific model (actual treatment) and the BCT-specific model (had they received baricitinib instead); the same dual stratification was applied to BCT-treated patients. The probabilities computed by the trained models were calibrated (Supplementary Figure 1) using the method that employs the Brier score decomposition (30). The threshold probability for each stratification was determined by maximizing the F1-score. Patients with a computed probability of Day-90 mortality under a specific immunomodulator exceeding the threshold were classified as high risk for the corresponding treatment.

Using this methodology, we classified the entire AMC critical COVID-19 cohort, including the development cohort and those excluded due to missing follow-up data, into four combinatorial risk subgroups: Group I (TCZ^LR^BCT^LR^) comprised patients with low risk for both immunomodulators; Group II (TCZ^LR^BCT^HR^) included patients at low risk for TCZ and high risk for BCT; Group III (TCZ^HR^BCT^LR^) contained patients at high risk for TCZ and low risk for BCT; and Group IV (TCZ^HR^BCT^HR^) comprised patients at high risk for both treatments. Next, we compared overall survival (OS) between BCT- and TCZ-treated patients within each group. Additionally, we classified the entire AMC critical COVID-19 cohort into two subgroups based solely on mortality risk for BCT or TCZ. Additionally, OS was compared between BCT and TCZ in the BCT^LR^ and TCZ^LR^ subgroups, respectively.

### 2.4 External validation of the models’ predictive performance

We evaluated model performance on the HYUMC test cohort, which included critical COVID-19 pneumonia patients treated with BCT or TCZ and with available Day-90 follow-up data. Predictive accuracy for Day-90 mortality was calculated using decision thresholds derived from the development cohort, optimized for the F1-score during cross-validation. Discrimination was assessed using the area under the receiver operating characteristic curve (ROC–AUC) and the area under the precision–recall curve (AUPRC), given the imbalance between survivors and non-survivors. For ROC–AUC, AUPRC, and accuracy, 95% confidence intervals (CIs) were estimated by stratified bootstrap resampling (1,000 replicates). Using the entire HYUMC critical COVID-19 cohort—including those without follow-up data, treated as right-censored—we estimated hazard ratios (HRs) to compare OS between BCT- and TCZ-treated patients within each risk subgroup. For these analyses, each patient was classified as low- or high-risk for each treatment (BCT and TCZ) according to the model-predicted probability of Day-90 mortality.

### 2.5 Statistical analysis

Survival analysis was performed using the Kaplan–Meier method, with differences in survival rates assessed via the log-rank test. Unlike model development, right-censored patient data were included in Kaplan–Meier analyses. The Cox proportional hazards model was employed to estimate HRs in multivariate analysis, with *p*-values calculated using the likelihood method. We verified the proportional hazards assumption using Schoenfeld residuals and found no violations. The *lifelines* package was used for Cox proportional hazards modeling. For subgroup comparisons, categorical and continuous variables were compared using chi-squared, Fisher’s exact tests, t-tests, or Mann–Whitney U tests, as appropriate. Statistical significance was defined as *p* < 0.05. All statistical analyses were conducted using the *SciPy* package, while Python (version 3.11.9) was used for implementation. For model performance, ROC–AUC, AUPRC, and accuracy were accompanied by 95% CIs derived from stratified bootstrap resampling with 1,000 replicates.

## 3 Results

### 3.1 Patient characteristics

**Figure 1:**
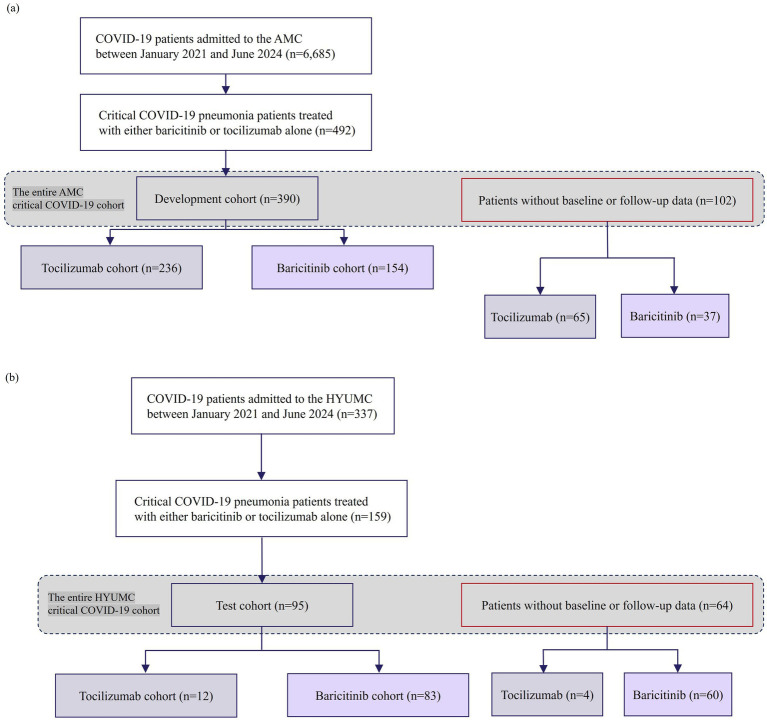
Study flow diagram showing the composition of **(a)** the development cohort and **(b)** the test cohort. The entire AMC critical COVID-19 cohort (*n* = 492) comprised the development cohort (*n* = 390) and patients without follow-up data (*n* = 102). The entire HYUMC critical COVID-19 cohort (*n* = 159) comprised the test cohort (*n* = 95) and patients without follow-up data (*n* = 64). Both entire critical COVID-19 cohorts were used for survival analyses of subgroups stratified by treatment-specific risk (patients without follow-up data treated as right-censored). COVID-19, Coronavirus disease-2019; AMC, Asan Medical Center; n, number; HYUMC, Hanyang University Medical Center.

Records of 6,388 COVID-19 patients admitted to AMC were reviewed; 492 critically ill patients treated with BCT or TCZ comprised the entire AMC critical COVID-19 cohort. After excluding 102 without baseline or follow-up data up to Day 90 post-initiation of immunomodulators, the development cohort included 390 patients (TCZ 236; BCT 154). The development cohort was further stratified into training and validation sets in a 4:1 ratio (Figure 1a). At HYUMC, 159 critically ill patients treated with BCT or TCZ were identified from 337 admissions and comprised the entire HYUMC critical COVID-19 cohort; excluding 64 without Day-90 data yielded a 95-patient test cohort (TCZ 12; BCT 83) (Figure 1b).

The demographic and clinical characteristics of the development and test cohorts are presented in Table 1. The test cohort includes only variables used in the model. The test cohort was older (median age 71), whereas the development cohort had more patients with solid malignancies or solid-organ transplants. Diastolic blood pressure (DBP), respiratory rate, and most laboratory values were comparable between the two cohorts. Within the development cohort, leukocyte count, hemoglobin, and creatinine did not differ between TCZ and BCT groups despite BCT contraindications in neutropenia, severe anemia, and chronic kidney disease (Supplementary Table 1).

**Table 1:** Demographic and clinical characteristics of the development and test cohorts.

Characteristic	Development cohort			Test cohort^a^		
	Tocilizumab cohort *n* = 236 (%)	Baricitinib cohort *n* = 154 (%)	Total *n* = 390 (%)	Tocilizumab cohort *n* = 12 (%)	Baricitinib cohort *n* = 83 (%)	Total *n* = 95 (%)
Age, years, median (range)	64 (21–80)	66 (24–80)	65 (21–80)	72 (27–80)	71 (29–80)	71 (27–80)
Male sex	152 (64)	96 (62)	248 (64)	6 (50)	46 (55)	52 (54)
Weight, kg, median (range)	67 (37–115)	63 (33–109)	65 (33–115)	60 (52–71)	64 (40–105)	65 (40–105)
Comorbidities						
Diabetes mellitus	31 (13)	8 (5)	39 (10)	–	–	–
Hypertension	9 (4)	5 (3)	14 (4)	–	–	–
Cardiovascular disease	29 (12)	19 (12)	48 (12)	–	–	–
Chronic kidney disease	50 (21)	51 (33)	101 (26)	–	–	–
Chronic lung disease	9 (4)	2 (1)	11 (3)	–	–	–
Chronic liver disease	7 (3)	7 (5)	14 (4)	–	–	–
Rheumatic disease	5 (2)	6 (4)	11 (3)	–	–	–
Solid malignancy	40 (17)	24 (16)	68 (17)	0 (0)	4 (5)	4 (4)
Hematologic malignancy	44 (19)	24 (16)	68 (17)	–	–	–
Solid organ transplantation	33 (14)	47 (31)	80 (21)	0 (0)	2 (2)	2 (1)
Baseline physiology, median (range; missing %)^b^						
Systolic blood pressure, mmHg	124 (95–171; 0%)	125 (97–160; 0%)	124 (95–171; 0%)	–	–	–
Diastolic blood pressure, mmHg	73 (48–96; 0%)	71 (55–92; 0%)	72 (48–96; 0%)	68 (56–87; 0%)	73 (56–99; 0%)	73 (56–99; 0%)
Heart rate, beats/min	82 (54–153; 0%)	80 (56–121; 0%)	82 (54–153; 0%)	86 (86–86; 92%)	91 (60–102; 90%)	89 (60–102; 91%)
Respiratory rate, breaths/min	22 (14–33; 1%)	20 (15–31; 0%)	21 (14–33; 1%)	21 (17–33; 0%)	21 (14–39; 5%)	21 (14–39; 4%)
Body temperature, °C	36.7 (34.6–37.6; 1%)	36.9 (36.1–38.4; 0%)	36.8 (34.6–38.4; 1%)	–	–	–
Peripheral oxygenation saturation, %	95 (82–100; 1%)	95 (82–100; 1%)	95 (82–100; 1%)	–	–	–
Laboratory data, mean (range; missing %)^b^						
Leukocyte, × 10^3^/uL	7.6 (0.1–39.4; 0%)	6.7 (0.1–14.4; 3%)	7.3 (0.1–39.4; 1%)	–	–	–
Hemoglobin, g/dL	12.1 (6.4–18.0; 0%)	10.8 (6.5–16.7; 3%)	11.4 (6.4–18.0; 1%)	11.2 (8.5–13.6; 0%)	12.4 (7.4–15.2; 2%)	12.2 (7.4–15.2; 2%)
Platelet, × 10^3^/uL	165.0 (9.0–568.0; 0%)	163.0 (17.0–451.0; 3%)	164.0 (9.0–568.0; 1%)	96.5 (47.0–399.0; 0%)	180.0 (25.0–455.0; 2%)	172.0 (25.0–455.0; 2%)
C-reactive protein, mg/dL	12.4 (0.1–59.7; 0%)	8.5 (0.1–31.7; 4%)	10.3 (0.1–59.7; 2%)	–	–	–
Aspartate aminotransferase, U/L	39 (12–325; 0%)	32 (11–313; 8%)	37 (11–325; 3%)	–	–	–
PT-INR	1.1 (0.9–3.9; 7%)	1.1 (0.9–2.6; 16%)	1.1 (0.9–3.9; 11%)	–	–	–
Procalcitonin, ng/mL	0.4 (0.06–29.2; 36%)	0.2 (0.07–36.5; 58%)	0.3 (0.06–28.2; 45%)	–	–	–
Creatinine, mg/dL	0.9 (0.2–11.7; 0%)	0.8 (0.3–10.5; 2%)	0.8 (0.2–11.7; 1%)	0.9 (0.3–1.9; 0%)	0.8 (0.2–7.0; 2%)	0.8 (0.2–7.0; 2%)
Albumin, g/dL	2.6 (1.7–4.1; 1%)	2.5 (1.4–3.6; 8%)	2.6 (1.4–4.1; 4%)	2.7 (1.9–3.6; 8%)	3.2 (2.1–4.0; 5%)	3.2 (1.9–4.0; 5%)
Potassium, mEq/L	4.2 (2.8–6.9; 0%)	4.2 (2.8–5.6; 2%)	4.2 (2.8–6.9; 1%)	4.1 (3.4–6.1; 8%)	4.0 (3.0–4.9; 2%)	4.0 (3.0–6.1; 3%)
Bicarbonate, mmol/L	21.7 (12.2–36.3; 9%)	22.6 (9.5–35.9; 21%)	22.4 (9.5–36.3; 14%)	–	–	–
Lactic acid, mmol/L	1.3 (0.6–5.2; 9%)	1.4 (0.6–3.0; 23%)	1.4 (0.6–5.2; 15%)	–	–	–
Blood urea nitrogen, mg/dL	21.5 (3.0–113.0; 0%)	20.0 (5.5–109.0; 3%)	20.5 (3.0–113.0; 1%)	21.3 (10.1–53.8; 0%)	21.5 (8.8–67.0; 2%)	21.4 (8.8–67.0; 2%)
pH	7.4 (7.3–7.5; 9%)	7.4 (7.2–7.5; 21%)	7.4 (7.2–7.5; 14%)	–	–	–

Data are presented as the number (%) of patients unless otherwise indicated. Abbreviations: n, number; PT-INR, prothrombin time-international normalized ratio. ^a^Data used in the ML models were obtained from the test cohort and features not used in the ML models were omitted. ^b^Baseline physiological measures and laboratory test results represent the average values recorded from 1 day prior to the initiation of immunomodulators to the day of initiation.

Mortality was higher in the test than in the development cohort and was consistently higher in TCZ- than BCT-treated patients (development: 28.4% vs. 23.4%; test: 50.0% vs. 34.1%). The proportion of patients receiving remdesivir was similar in the entire AMC (*n* = 492) and HYUMC (*n* = 159) critical COVID-19 cohorts (88.3 and 85.7%, respectively). Similarly, corticosteroid use, such as dexamethasone and methylprednisolone, was comparable between the two centers (87.6% vs. 86.4%, respectively).

### 3.2 ML-based mortality prediction and risk stratification

**Figure 2:**
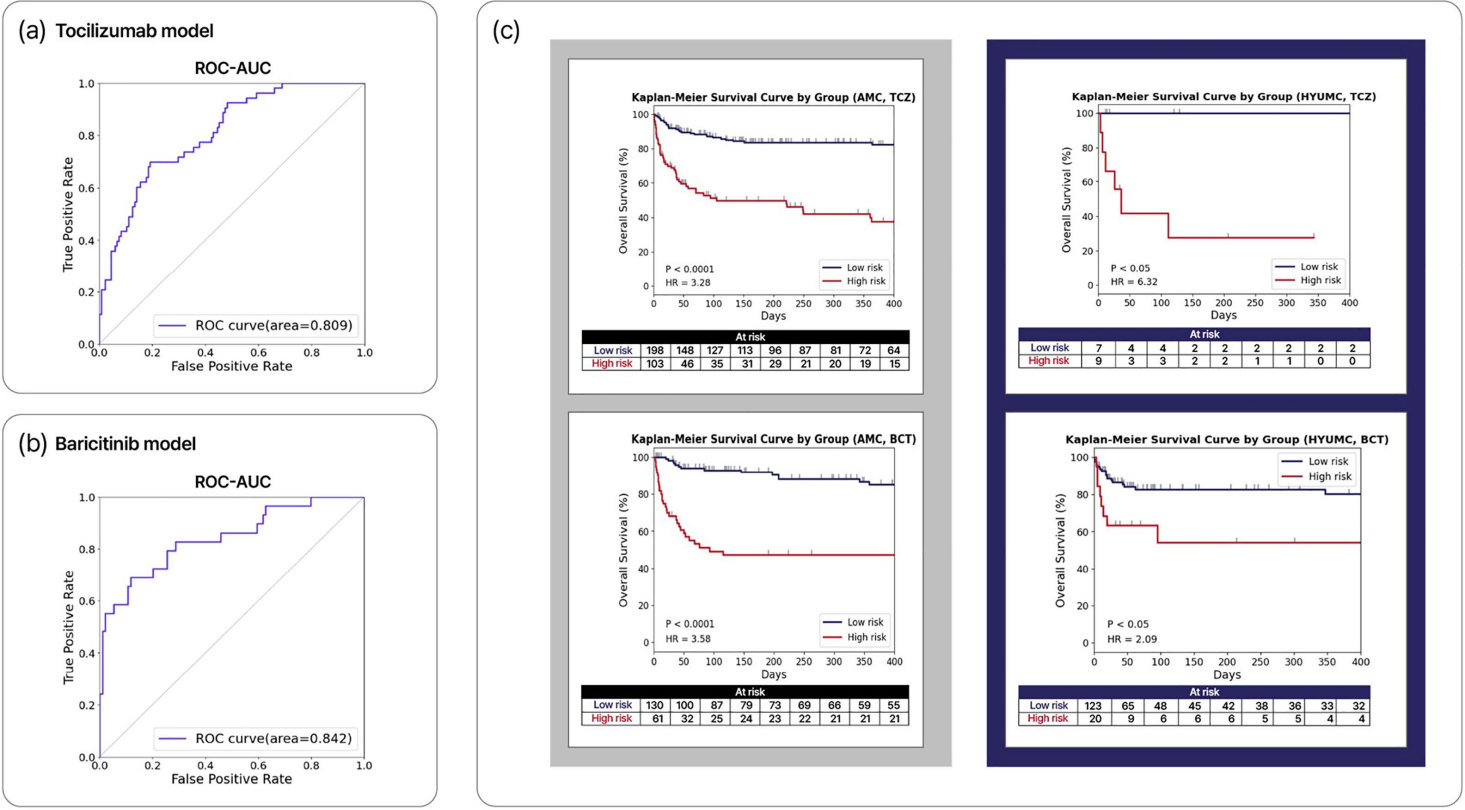
ML models and treatment-specific risk stratification. **(a)** ROC-AUC of the ML models predicting Day-90 mortality in the training set of the development cohort for patients receiving TCZ (*n* = 188) or **(b)** BCT (*n* = 123). **(c)** Survival curves based on the risk stratification using the ML models (TCZ- and BCT-specific models shown at the top and bottom, respectively). The left panel depicts results from the entire AMC critical COVID-19 cohort (*n* = 492). The right panel depicts results from the entire HYUMC critical COVID-19 cohort (*n* = 159). ROC-AUC, Receiver Operating Characteristic-Area Under the Curve.

The development cohort (*n* = 390) was split into training (*n* = 311) and internal validation (*n* = 79) sets in a 4:1 ratio. Separate ML models were trained to predict Day-90 mortality for TCZ-treated (*n* = 188) and BCT-treated (*n* = 123) patients using the training set. Recursive feature elimination with cross-validation (RFECV) identified eight features with the highest predicted performances in both TCZ and BCT ML models (Supplementary Figure 2). For TCZ model, key covariates were age, weight, DBP, blood urea nitrogen (BUN), creatinine, hemoglobin, platelets, and albumin to predict Day-90 mortality in TCZ-treated patients, achieving an ROC-AUC of 0.81 (95% CI, 0.74–0.87) during internal validation (Figure 2a). The accuracy was 0.67 when applied to the TCZ-treated patients (*n* = 48) in the internal validation set. The AUPRC in the internal validation set was 0.65 (95% CI, 0.52–0.77), and the precision–recall curve is shown in Supplementary Figure 3. Similarly, for BCT model, age, weight, heart rate, respiratory rate, potassium, serum creatinine, hemoglobin, and albumin were selected to predict Day-90 mortality in BCT-treated patients, achieving an ROC-AUC of 0.84 (95% CI, 0.75–93) and AUPRC of 0.74 (95% CI, 0.57–0.87) during internal validation (Figure 2b). Accuracy was 0.77 when applied to the BCT-treated patients (*n* = 31) in the internal validation set. Model performance did not vary significantly with the way missing values were handled (Supplementary Table 3).

In the development cohort, baseline clinical characteristics and differences between TCZ^HR^ and TCZ^LR^ (among TCZ-treated patients) and between BCT^HR^ and BCT^LR^ (among BCT-treated patients) are summarized in Tables 2, 3. In the TCZ^HR^ subgroup, the median age, proportions of patients with chronic kidney disease or malignancies, and levels of procalcitonin, lactic acid, and BUN were significantly higher compared to the TCZ^LR^ subgroup. Conversely, the median weight, DBP, and hemoglobin, platelets, albumin, and bicarbonate levels were all significantly lower in the TCZ^HR^ subgroup compared to the TCZ^LR^ subgroup.

**Table 2:** Baseline clinical characteristics of the TCZ-specific risk subgroups.

Characteristic	TCZ^HR^ *n* = 46 (%)	TCZ^LR^ *n* = 190 (%)	*P*-value
Age, years, median (range)	73 (66–76)	61 (51–66)	<0.001
Male sex	46 (57)	106 (68)	0.104
Weight, kg, median (range)	60 (52–70)	69 (60–80)	<0.001
Comorbidities			
Diabetes mellitus	10 (12)	21 (14)	0.955
Hypertension	4 (5)	5 (3)	0.769
Cardiovascular disease	13 (16)	16 (10)	0.288
Chronic kidney disease	25 (31)	25 (16)	0.014
Chronic lung disease	3 (4)	6 (4)	1.000
Chronic liver disease	3 (4)	4 (3)	0.937
Rheumatic disease	4 (5)	1 (1)	0.089
Solid malignancy	24 (30)	16 (10)	<0.001
Hematologic malignancy	25 (31)	19 (12)	<0.001
Solid organ transplantation	15 (19)	18 (12)	0.210
Baseline physiology, median (range)^a^			
Systolic blood pressure, mmHg	123 (115–133)	122 (113–132)	0.345
Diastolic blood pressure, mmHg	65 (61–73)	70 (64–77)	0.004
Heart rate, beats/min	85 (76–101)	82 (72–97)	0.127
Respiratory rate, breaths/min	22 (20–25)	23 (20–26)	0.480
Body temperature, °C	36.9 (36.5–37.2)	36.9 (36.5–37.2)	0.839
Peripheral oxygenation saturation, %	96 (94–96)	96 (94–97)	0.577
Laboratory data, median (range)^a^			
Leukocyte, × 10^3^/uL	8.0 (4.3–11.8)	7.6 (5.1–11.3)	0.961
Hemoglobin, g/dL	9.3 (8.3–10.8)	12.8 (11.1–14.1)	<0.001
Platelet, × 10^3^/uL	123 (66.0–172)	190 (133–245)	<0.001
C-reactive protein, mg/dL	12.7 (6.5–17.7)	12.2 (7.7–19.0)	0.698
Aspartate aminotransferase, U/L	40.0 (29.5–59.5)	38.2 (30.6–57.5)	0.902
PT-INR	1.1 (1.0–1.3)	1.1 (1.0–1.2)	0.002
Procalcitonin, ng/mL	0.7 (0.2–2.3)	0.3 (0.1–0.8)	0.004
Creatinine, mg/dL	1.0 (0.7–2.0)	0.8 (0.7–1.3)	0.136
Albumin, g/dL	2.4 (2.1–2.7)	2.7 (2.4–3.1)	<0.001
Potassium, mEq/L	4.2 (3.8–4.7)	4.2 (3.9–4.6)	0.981
Bicarbonate, mmol/L	20.8 (17.8–23.6)	22.7 (20.3–24.6)	0.005
Lactic acid, mmol/L	1.6 (1.2–2.2)	1.3 (1.1–1.8)	0.011
Blood urea nitrogen, mg/dL	27.5 (18.0–51.5)	19.0 (12.5–28.9)	<0.001
pH	7.4 (7.4–7.5)	7.4 (7.4–7.5)	0.865

Data are presented as the number (%) of patients unless otherwise indicated. Abbreviations: TCZ, tocilizumab; HR, high-risk; LR, low-risk; n, number; PT-INR, prothrombin time-international normalized ratio. ^a^Baseline physiological measures and laboratory test results represent the average values recorded from one day prior to the initiation of immunomodulators to the day of initiation.

**Table 3:** Baseline clinical characteristics of the BCT-specific risk subgroups.

Characteristic	BCT^HR^ *n* = 29 (%)	BCT^LR^ *n* = 125 (%)	*P*-value
Age, years, median (range)	69 (61–75)	65 (56–69)	0.007
Male sex	35 (65)	61 (61)	0.770
Weight, kg, median (range)	59 (50–66)	64 (55–72)	0.018
Comorbidities			
Diabetes mellitus	4 (7)	4 (4)	0.597
Hypertension	2 (4)	3 (3)	1.000
Cardiovascular disease	13 (24)	6 (6)	0.003
Chronic kidney disease	15 (28)	36 (36)	0.392
Chronic lung disease	0 (0)	2 (2)	0.764
Chronic liver disease	4 (7)	3 (3)	0.397
Rheumatic disease	3 (6)	3 (3)	0.730
Solid malignancy	18 (33)	11 (11)	0.002
Hematologic malignancy	10 (19)	14 (14)	0.614
Solid organ transplantation	14 (26)	33 (33)	0.468
Baseline physiology, median (range)^a^			
Systolic blood pressure, mmHg	121 (110–132)	127 (113–141)	0.136
Diastolic blood pressure, mmHg	70 (62–77)	74 (65–82)	0.131
Heart rate, beats/min	93 (81–105)	78 (70–86)	0.001
Respiratory rate, breaths/min	22 (19–25)	20 (18–22)	0.001
Body temperature, °C	36.8 (36.6–37.2)	36.8 (36.5–37.3)	0.588
Peripheral oxygenation saturation, %	96 (94–97)	96 (95–97)	0.311
Laboratory data, median (range)^a^			
Leukocyte, × 10^3^/uL	6.7 (3.5–10.1)	6.6 (4.6–9.7)	0.754
Hemoglobin, g/dL	8.9 (7.8–10.6)	11.7 (10.0–13.3)	<0.001
Platelet, × 10^3^/uL	129 (72.8–200)	184 (133–237)	0.003
C-reactive protein, mg/dL	11.0 (6.3–16.3)	7.3 (3.4–10.7)	0.001
Aspartate aminotransferase, U/L	31.5 (22.2–53.5)	30.5 (20.3–51.0)	0.631
PT-INR	1.1 (1.0–1.4)	1.1 (1.0–1.2)	0.013
Procalcitonin, ng/mL	0.4 (0.1–1.5)	0.2 (0.1–0.5)	0.098
Creatinine, mg/dL	0.7 (0.5–1.2)	0.9 (0.7–1.4)	0.002
Albumin, g/dL	2.2 (1.9–2.4)	2.8 (2.5–3.0)	<0.001
Potassium, mEq/L	4.0 (3.8–4.3)	4.2 (3.9–4.5)	0.083
Bicarbonate, mmol/L	23.2 (20.3–25.8)	22.2 (18.1–24.8)	0.089
Lactic acid, mmol/L	1.4 (1.0–2.0)	1.2 (1.0–1.7)	0.161
Blood urea nitrogen, mg/dL	19.2 (14.2–28.2)	20.7 (14.9–31.5)	0.627
pH	7.5 (7.4–7.5)	7.4 (7.4–7.5)	0.090

Data are presented as the number (%) of patients unless otherwise indicated. BCT, baricitinib; HR, high-risk; LR, low-risk; n, number; PT-INR, prothrombin time-international normalized ratio. ^a^Baseline physiological measures and laboratory test results represent the average values recorded from 1 day prior to the initiation of immunomodulators to the day of initiation.

Similarly, in the BCT^HR^ subgroup, the median age, proportions of patients with cardiovascular disease or solid malignancies, heart rate, respiratory rate, and C-reactive protein levels were significantly higher than in the BCT^LR^ subgroup, while median weight and levels of hemoglobin, platelets, and albumin were significantly lower. Severe comorbidities, including malignancies and solid organ transplantation, are well known to significantly impact overall mortality (31). In multivariable analyses, the ML-estimated risk was the strongest predictor of OS, compared to other features, including severe comorbidities (Supplementary Figure 4).

Applying stratification based on the TCZ- and BCT-specific model–estimated survival probabilities to the entire AMC cohort, we classified TCZ-treated patients (*n* = 301) as TCZ^HR^ (*n* = 103) or TCZ^LR^ (*n* = 198) and BCT-treated patients (*n* = 191) as BCT^HR^ (*n* = 61) or BCT^LR^ (*n* = 130). Overall mortality was significantly higher in the high-risk groups than in their corresponding low-risk groups (TCZ^HR^ vs. TCZ^LR^: HR 3.28, 95% CI 2.41–4.48, *p* < 0.001; BCT^HR^ vs. BCT^LR^: HR 3.58, 95% CI 2.39–5.37, *p* < 0.001). In the HYUMC cohort, BCT-treated patients (n = 143) showed an HR of 2.09 (95% CI 1.06–4.12; *p* = 0.016) between high- and low-risk groups. Among TCZ-treated patients (*n* = 16), the HR was 6.32 (95% CI, 3.18–12.60; *p* = 0.018), but the small sample size limits the interpretability of this result (Figure 2c).

### 3.3 Combinatorial risk stratification

**Figure 3:**
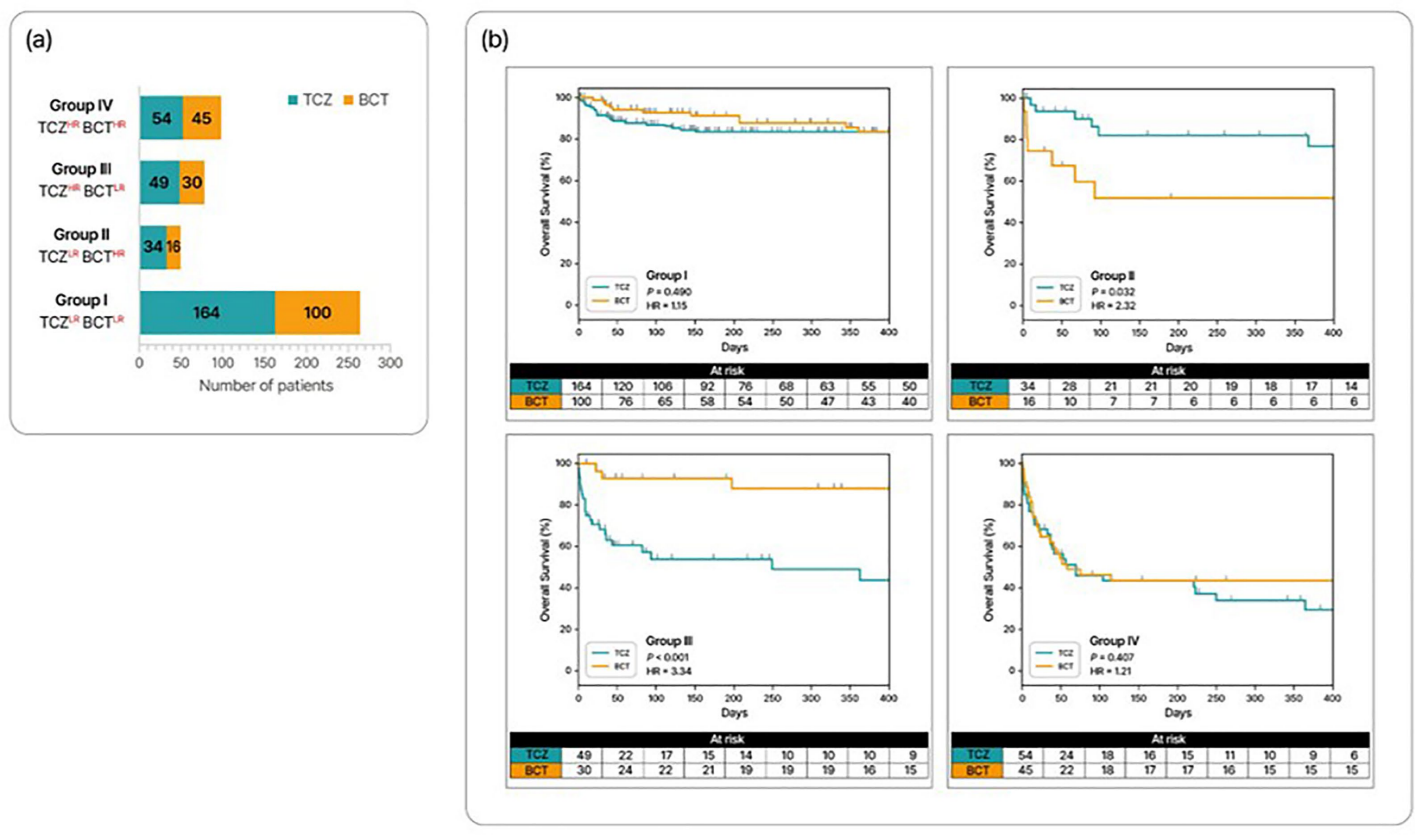
Combinatorial risk stratification of the entire AMC critical COVID-19 cohort (*n* = 492), based on risks of TCZ and BCT. **(a)** Number of patients in the four combinatorial risk subgroups. **(b)** Survival curves for patients in each subgroup treated with TCZ or BCT. HR, high-risk; LR, low-risk.

Using combinatorial risk stratification, we classified the entire AMC critical COVID-19 cohort (*n* = 492) into the four groups: Group I (*n* = 264), Group II (*n* = 50), Group III (*n* = 79), and Group IV (*n* = 99) (Figure 3a). Overall mortality did not differ between TCZ- and BCT-treated patients in Group I (HR 1.15, 95% CI 0.79–1.66; *p* = 0.490) or Group IV (HR 1.21, 95% CI 0.78–1.89; *p* = 0.407). In Group II, BCT-treated patients had significantly higher mortality than TCZ-treated patients (HR 2.32, 95% CI 1.05–5.13; *p* = 0.032), whereas in Group III, TCZ-treated patients had significantly higher mortality than BCT-treated patients (HR 3.34, 95% CI 1.94–5.76; *p* < 0.001) (Figure 3b). Because Group II had relatively few patients and an imbalance in the number of TCZ- and BCT-treated cases, the observed excess mortality with BCT should be interpreted cautiously. The HYUMC cohort (*n* = 159) showed similar directions of effect, although Group II was underpowered due to small sample size (Supplementary Figure 5).

Although ML models are prognostic only and cannot definitively determine counterfactual outcomes with the alternative immunomodulator, these findings suggest that model-guided choice might have improved the outcome in 65/492 patients (13.2%): 16 BCT-treated patients in Group II and 49 TCZ-treated patients in Group III. Among those who received an immunomodulator despite being at high risk for that specific agent, 49/103 TCZ^HR^ patients treated with TCZ (47.6%) and 16/61 BCT^HR^ patients treated with BCT (26.2%) might have had different outcomes with the alternative agent.

Additionally, using risk stratification based solely on mortality risk for BCT or TCZ, 70% of patients in the entire AMC critical COVID-19 cohort (*n* = 492) were categorized into the BCT^LR^ subgroup (*n* = 343) and 64% into the TCZ^LR^ subgroup (*n* = 314). In the BCT^LR^ subgroup, TCZ-treated patients had significantly higher mortality than BCT-treated patients (HR 1.61, 95% CI 1.18–2.18; *p* = 0.007) (Supplementary Figure 6A). In the TCZ^LR^ subgroup, mortality was similar (HR 1.05, 95% CI 0.74–1.49; *p* = 0.786). Similar trends were observed in the BCT^LR^ subgroup (*n* = 135) and the TCZ^LR^ subgroup (*n* = 111) from the HYUMC critical COVID-19 cohort (*n* = 159), although no significant difference in overall mortality was detected in the BCT^LR^ or TCZ^LR^ subgroups due to the limited number of TCZ-treated patients (Supplementary Figure 6B).

## 4 Discussion

In this study, we developed and externally validated two separate machine-learning models to predict Day-90 mortality in critically ill COVID-19 patients treated with BCT or TCZ. Using only readily available clinical variables, the models achieved acceptable discrimination (ROC-AUC 0.81–0.84 in internal validation) and identified distinct risk profiles for each immunomodulator. When combinatorial risk stratification was applied to the entire AMC cohort (*n* = 492), 13.2% of patients received an immunomodulator for which they were classified as high-risk while being low-risk for the other agent. Survival analyses suggested that model-guided selection of the lower-risk agent in these patients could have altered outcomes, although these models are prognostic rather than causal and counterfactual outcomes under the alternative therapy cannot be definitively established. These findings provide the first treatment-specific prognostic framework for choosing between BCT and TCZ in critically ill patients requiring HFNC, NIV, MV, or ECMO.

Previous randomized and observational studies comparing BCT and TCZ have reported conflicting results, with some showing similar efficacy, others favoring BCT in invasively ventilated patients, and still others demonstrating non-inferiority of BCT (12, 13, 15, 32). However, these studies compared overall treatment effects and focused on specific subgroups of patients with severe or critical COVID-19 pneumonia (e.g., MV/ECMO or HFNC/NIV patients). In contrast, our treatment-specific modeling approach, applied across the full spectrum of critically ill COVID-19 pneumonia patients, reveals that the relative benefit of BCT versus TCZ is dependent on baseline clinical characteristics. This framework explains much of the apparent discrepancy across prior studies and provides a practical method for personalizing immunomodulator selection.

The clinical features selected by our ML models were highly consistent with established prognostic factors in critical illness. Both TCZ and BCT models included age, weight, creatinine, hemoglobin, and albumin as clinical variables. In the TCZ model, older age, elevated BUN, and thrombocytopenia were significantly associated with higher mortality. In the BCT model, older age, tachycardia, tachypnea, anemia, and hypoalbuminemia were significantly associated with higher mortality (Supplementary Figure 7). These features align closely with components of validated severity scores [e.g., SOFA platelet component (33); NEWS/NEWS2 heart and respiratory rates (34, 35)] and known prognostic markers such as age, BUN, anemia, and hypoalbuminemia (36–39). The observed differences between the two models likely reflect their distinct pharmacologic mechanisms of action and the heterogeneous inflammatory phenotypes encountered in critical COVID-19.

Notably, BCT conferred a significant survival benefit only in patients classified as low-risk for BCT (BCT^LR^ subgroup), whereas no corresponding benefit was observed for TCZ in the TCZ^LR^ subgroup. In the external test cohort, differences in overall survival between the two low-risk subgroups did not reach statistical significance, likely because of the very small number of TCZ-treated patients (*n* = 12). These observations do not imply overall superiority of BCT but rather underscore the presence of heterogeneous treatment effects that can be captured by treatment-specific risk models.

Treatment assignment was non-randomized and confounding by contraindications was inevitable. Patients with active bacterial or fungal co-infection, profound neutropenia, inability to swallow tablets, or advanced chronic kidney disease were systematically directed toward TCZ owing to BCT contraindications or practical considerations, whereas suspected secondary infection favored BCT, because TCZ is relatively contraindicated in that setting. This bidirectional selection bias almost certainly contributed to the higher crude mortality observed in the TCZ-treated patients than in the BCT-treated patients.

Mechanistically, the broader cytokine-suppressive profile of BCT may offer theoretical advantages in certain inflammatory phenotypes. TCZ selectively blocks the IL-6 receptor (40), whereas BCT inhibits JAK1/JAK2 signaling and thereby interferes with multiple cytokine pathways, including IL-6, IL-2, IL-15, and interferon signaling (41). This wider spectrum could, in principle, sustain anti-inflammatory effects more effectively over several days of treatment. Supporting this hypothesis, a previous study in mechanically ventilated or ECMO-supported patients reported lower Day-30 mortality with BCT than with TCZ (32), and other retrospective analyses observed greater respiratory improvement at Day 7 or survival with BCT in severe-to-critical COVID-19 (14, 15). However, these observational comparisons are subject to the same confounding by indication and randomized data directly comparing the two agents remain limited.

Our goal was to build ML models that predict mortality using a minimal, clinically practical set of covariates. ML can capture complex, non-intuitive relations from existing clinical data, making it more feasible than biomarker-heavy, multi-omic approaches that demand substantial time and resources (42). Although ROC–AUC can rise with more weakly correlated features, we intentionally selected only the most predictive variables for Day-90 mortality in critical COVID-19, and we show how these features contribute to model probabilities for each therapy (Supplementary Figure 8). Using too many features hampers interpretability, increases data-collection burden and cost, and heightens overfitting risk, reducing generalizability (43). Because there is no single gold standard for feature selection, the number and composition of features should be guided by predictive performance and dataset size (44). We trained on the largest available development cohort for each immunomodulator, and external validation in the test cohort showed similar performance.

Despite these strengths, our study has limitations. First, the models were developed and validated in single-center cohorts, and temporal shifts in circulating variants (Alpha → Delta → Omicron), as well as staggered approvals of BCT/TCZ, may limit generalizability. Therefore, multi-center validation across various settings and pandemic phases is needed. Second, there was substantial treatment-arm imbalance—especially in the test cohort (TCZ *n* = 12 vs. BCT *n* = 83)—which reduces power for subgroup/survival comparisons and may bias effect estimates; results should be interpreted with caution and larger, more balanced cohorts are warranted. Third, the retrospective design entails potential selection bias and uneven baseline severity between treatment arms. Additionally, we analyzed only critically ill patients receiving immunomodulators, limiting applicability to patients with moderate/severe disease not receiving these agents. Fourth, SARS-CoV-2 vaccination status was not included. While vaccination significantly influences the severity of COVID-19, its impact on the effectiveness of immunomodulators in critically ill patients remains uncertain. Fifth, our ML models used static (not time-series) features due to missing temporal data in the test cohort; despite this, discrimination was moderate and static-feature models are more practical for bedside use. Finally, we do not treat between-model differences for the same patient as counterfactual effects because treatment was non-randomized and models were trained separately; isotonic regression was used only for within-arm calibration. Accordingly, findings are hypothesis-generating and require confirmation in prospective randomized trials or rigorously adjusted observational studies under a shared covariate structure.

## 5 Conclusion

Despite these limitations, to the best of our knowledge, this study is the first to develop and externally validate two treatment-specific prognostic machine-learning models for critically ill patients receiving either baricitinib or tocilizumab. These trained ML models can be a useful adjunct in selecting immunomodulators for critically ill COVID-19 pneumonia patients. Further studies are needed to investigate the potential application of these models in enhancing survival outcomes.

## Data Availability

The raw data supporting the conclusions of this article will be made available by the authors, without undue reservation.
