# Supplementary material for: Machine learning-based approach to guide the choice between baricitinib and tocilizumab in critical COVID-19 pneumonia treatment: a retrospective cohort study
Source: Front Med (Lausanne). 2026 Jan 7;12:1734109. doi: 10.3389/fmed.2025.1734109 (PMC12819291; doi:10.3389/fmed.2025.1734109)
Supplement: Supplementary file 1 [file Data_Sheet_1.docx]

**Supplementary Materials for**

**Machine learning-based approach to guide the choice between baricitinib and tocilizumab in critical COVID-19 pneumonia treatment: a retrospective cohort study**

Euijin Chang^1,*^, Myung-Soo Kim^1,2,*^, Se Yoon Park^3,*^, Kyungsup Kwon^1^, Hyeon Mu Jang^1^, So Yun Lim^1^, Seongman Bae^1^, Jiwon Jung^1^, Min Jae Kim^1^, Yong Pil Chong^1^, Sang-Oh Lee^1^, Sang-Ho Choi^1^, Yang Soo Kim^1^, Gyucheol Choi^2^, Sungwon Lim^4^, Jamin Koo^2,4,5,†^, Sung-Han Kim^1,†^

^1^Department of Infectious Diseases, Asan Medical Center, University of Ulsan College of Medicine, Seoul, Republic of Korea

^2^ImpriMedKorea, Inc., Seoul 03920, Republic of Korea

^3^Department of Internal Medicine, Hanyang University College of Medicine, Seoul, Republic of Korea

^4^ImpriMed, Inc., Mountain View, CA 94043, U.S.A.

^5^Department of Chemical Engineering, Hongik University, Seoul 04066, Republic of Korea

*These authors contributed equally to this manuscript.

^†^These authors contributed equally to this manuscript as senior authors.

**Table of Contents**

**Supplementary Figure 1**. Sliding-window calibration graph of predicted probability of positive response vs percentage of patients experiencing a positive response (window size = 5% of sample size; blue line = prediction; shaded region = 95% prediction interval; dotted line = perfect calibration) for (A) tocilizumab and (B) baricitinib.

**Supplementary Figure 2**. Recursive feature elimination with cross-validation demonstrating the highest predicting performance in both (A) TCZ and (B) BCT models when using eight features.

**Supplementary Figure 3.** Precision–recall curves and area under the precision–recall curve (AUPRC) for (**A**) TCZ- and (**B**) BCT-specific models applied to the internal validation set (n=79).

**Supplementary Figure 4**. Hazard ratios of the baseline characteristics including severe comorbidities for overall survival (OS) of the COVID-19 patients treated by (A) TCZ or (B) BCT as the first-line treatment.

**Supplementary Figure 5**. Combinatorial risk stratification of the test cohort based on mortality risks using TCZ and BCT.

**Supplementary Figure 6**. Survival curves for patients stratified into two subgroups for each immunomodulator based solely on overall mortality risk when using BCT or TCZ (A) in the development cohort and (B) in the test cohort.

**Supplementary Figure 7**. Forest plot for hazard ratios of the selected covariates in the TCZ and BCT ML models.

**Supplementary Figure 8**. SHAP analysis of the ML-generated probabilities for patients from Group I.

**Supplementary Table 1.** Contraindications for BCT and TCZ.

**Supplementary Table 2**. Proportions of missing values across the 38 input variables in the development cohort.

**Supplementary Table 3.** Performance of machine learning models predicting treatment outcome of the severe COVID-19 with respect to imputation of missing values.

**Supplementary Methods**.


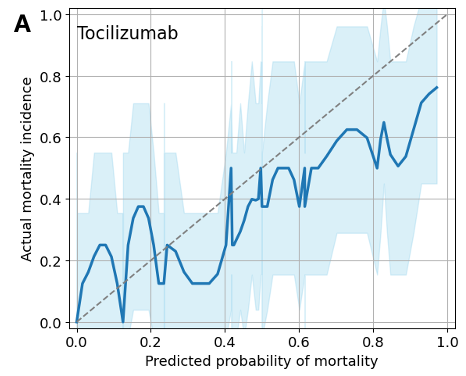

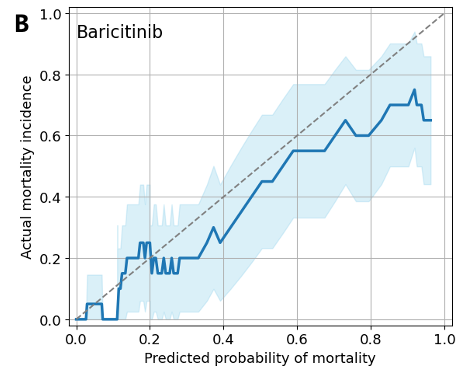


**Supplementary Figure 1**. Sliding-window calibration graph of predicted probability of positive response vs percentage of patients experiencing a positive response (window size = 5% of sample size; blue line = prediction; shaded region = 95% prediction interval; dotted line = perfect calibration) for (A) tocilizumab and (B) baricitinib.


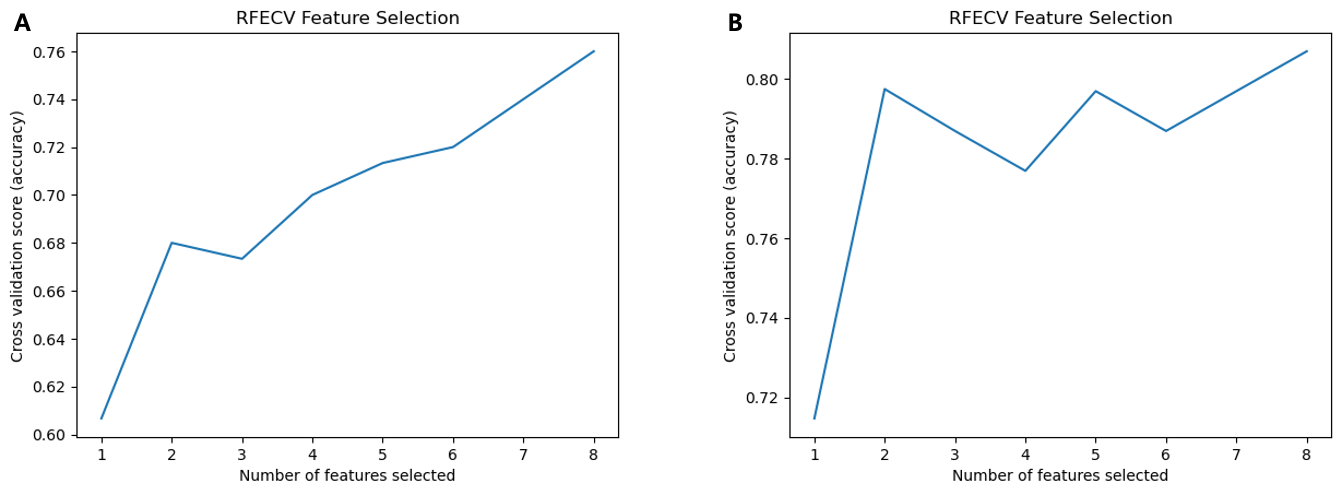


**Supplementary Figure 2**. Recursive feature elimination with cross-validation demonstrating the highest predicting performance in both (A) TCZ and (B) BCT models when using eight features.

Abbreviations: TCZ, tocilizumab; BCT, baricitinib; RFECV, recursive feature elimination with cross-validation.

**
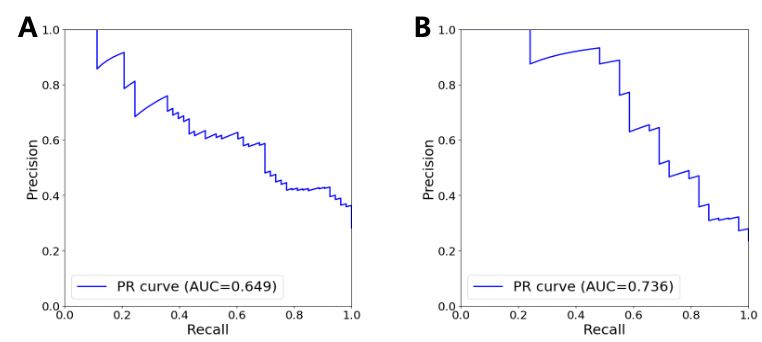
**

**Supplementary Figure 3.** Precision–recall curves and area under the precision–recall curve (AUPRC) for (**A**) TCZ- and (**B**) BCT-specific models applied to the internal validation set (n=79).

Abbreviations: TCZ, tocilizumab; BCT, baricitinib; PR, precision recall; AUC, area under the curve.

**
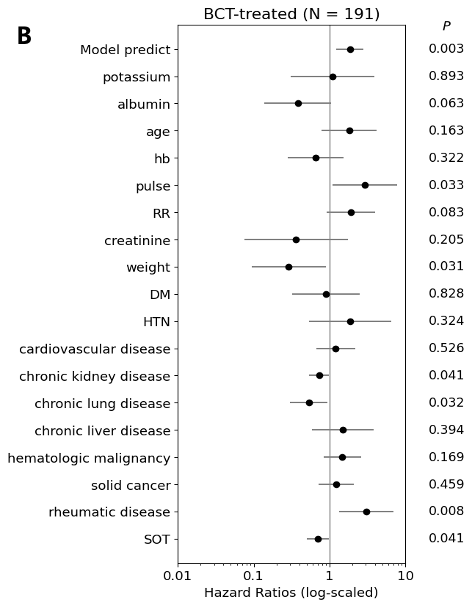
**


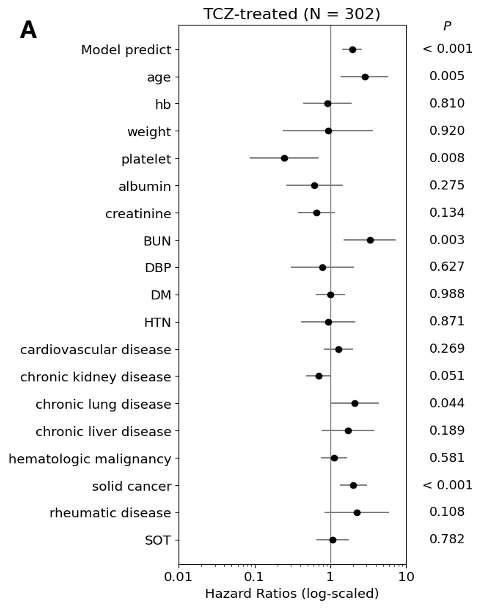


TCZ-treated (N = 301)

**Supplementary Figure 4**. Hazard ratios of the baseline characteristics including severe comorbidities for overall survival (OS) of the COVID-19 patients treated by (A) TCZ or (B) BCT as the first-line treatment. The HR values were calculated via the Cox proportional hazard modeling. This analysis was conducted for the entire AMC critical COVID-19 cohort (n=492).

Abbreviations: TCZ, tocilizumab; BCT, baricitinib; N, number; BUN, blood urea nitrogen; DBP, diastolic blood pressure; Hb, hemoglobin; RR, respiratory rate; SOT, solid organ transplantation.


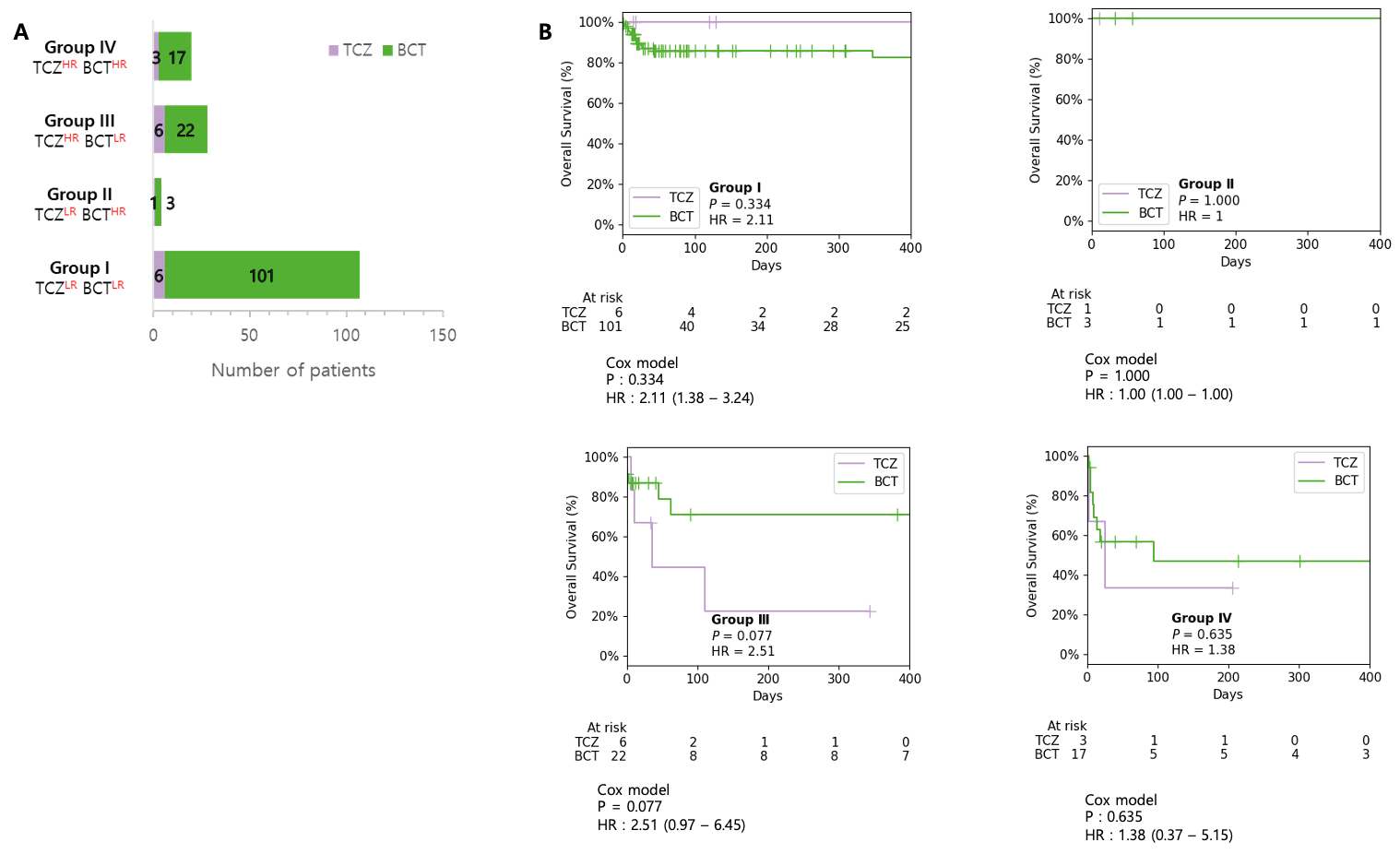


**Supplementary Figure 5.** Combinatorial risk stratification of the entire HYUMC critical COVID-19 cohort (n=159), based on mortality risks using TCZ and BCT. (A) Number of patients in the four combinatorial risk subgroups. (B) Survival curves for patients in each subgroup treated with TCZ or BCT.

Abbreviations: HR, high-risk; LR, low-risk; HR, hazard ratio


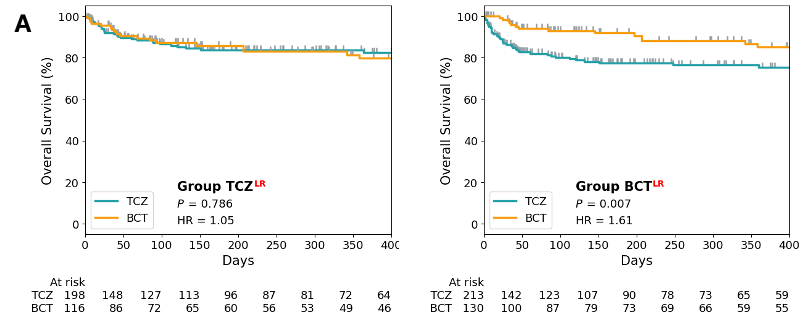


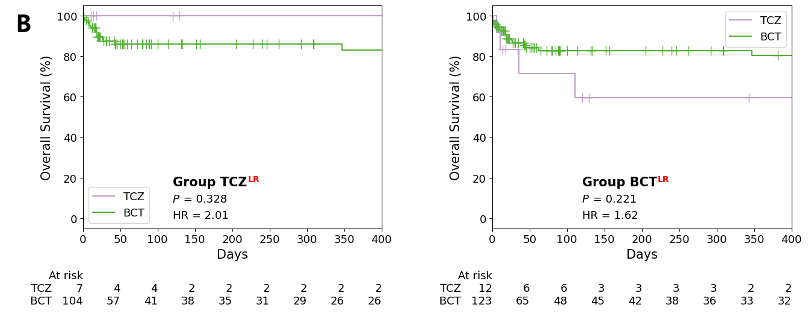


**Supplementary Figure 6.** Survival curves for patients stratified into two subgroups for each immunomodulator based solely on overall mortality risk when using BCT or TCZ. (A) In the entire AMC critical COVID-19 cohort (n=492), 314 and 343 patients formed the TCZ^LR^ and BCT^LR^ subgroups, respectively. (B) In the entire HYUMC critical COVID-19 cohort (n=159), 111 and 135 patients comprised the TCZ^LR^ and BCT^LR^ subgroups, respectively.

Abbreviation: LR, low-risk


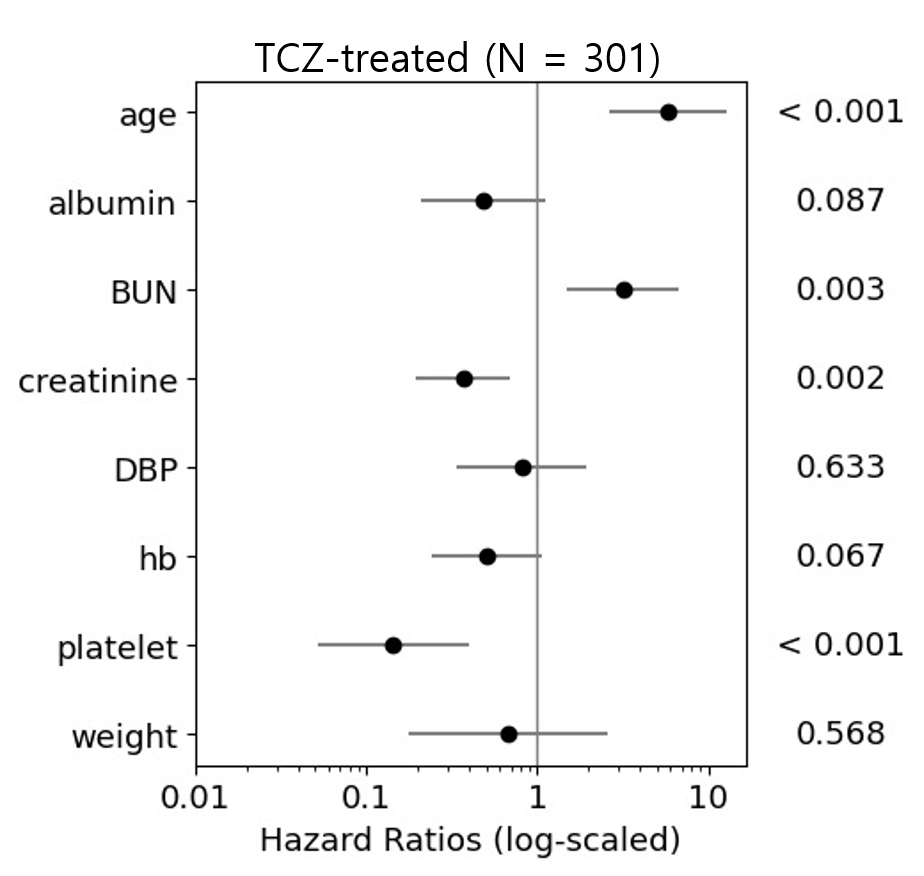

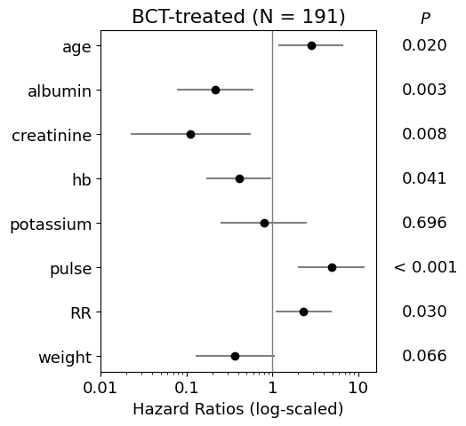


**Supplementary Figure 7**. Forest plot for hazard ratios of the selected covariates in the TCZ and BCT ML models.

Abbreviations: TCZ, tocilizumab; BCT, baricitinib; N, number; BUN, blood urea nitrogen; DBP, diastolic blood pressure; Hb, hemoglobin; RR, respiratory rate

**
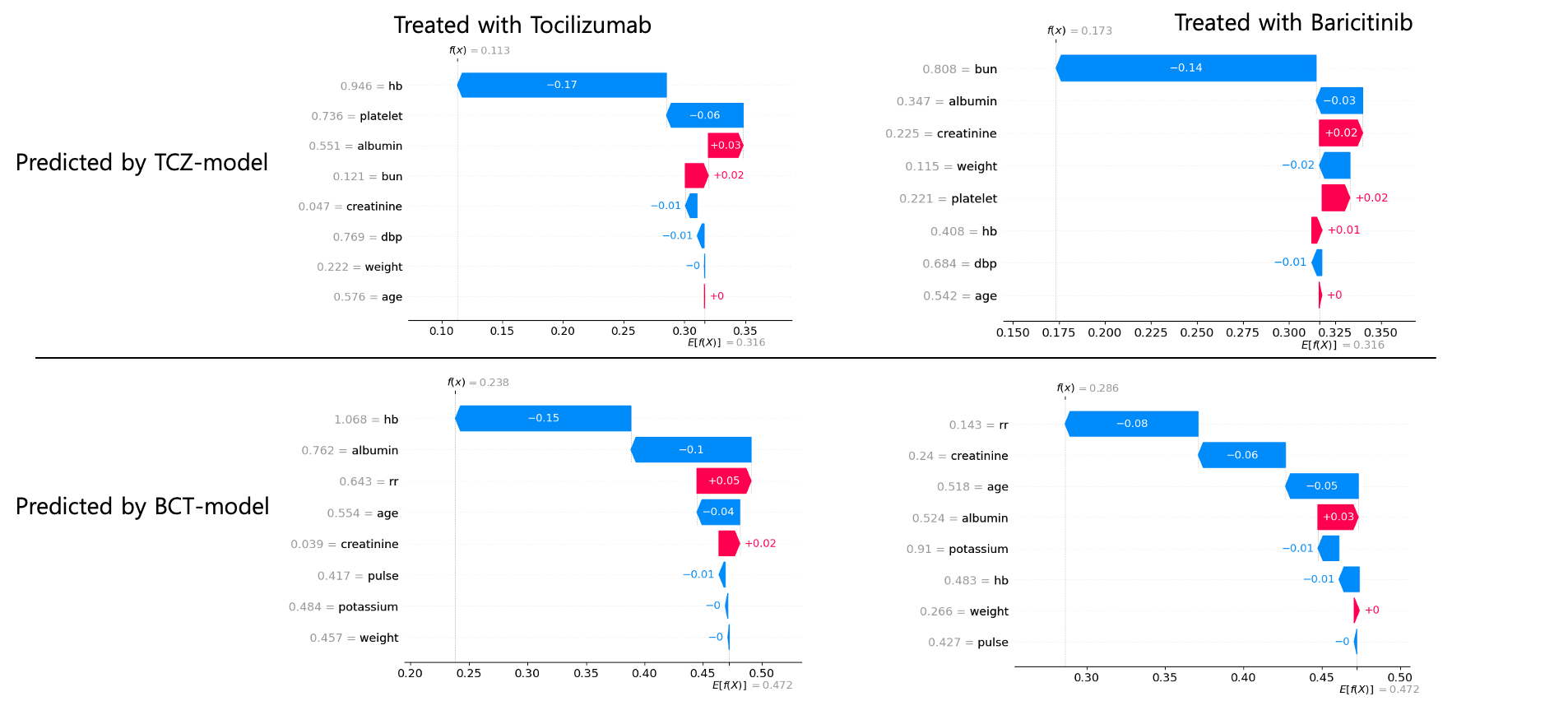
**

**Supplementary Figure 8**. SHAP analysis of the ML-generated probabilities for a patient from Group I. *f(X)* represents the model-generated probability of mortality for TCZ (left) and BCT (right). Numbers inside horizontal bars denote negative (blue) or positive (red) changes in the baseline probability, E[f(X)], driven by the covariate (e.g., creatinine) used by the ML model. The numbers in grey represent the specific value for the patient described in this analysis.

**Supplementary Table 1.** Contraindications for BCT and TCZ.

| **Immunomodulator** | **Contraindications** |
| --- | --- |
| **BCT** | Severe active infections, including untreated or inadequately treated hepatitis, HIV, fungal infections, and active tuberculosis |
|  | Chronic kidney disease with a GFR <30 mL/min/1.73m^2^ or end-stage renal disease |
|  | Severe anemia (hemoglobin <8 mg/dL) |
|  | Lymphopenia (absolute lymphocyte count <500/mm^3^) |
|  | Neutropenia (absolute neutrophil count <1,000/mm^3^) |
| **TCZ** | Hypersensitivity reaction to the tocilizumab dosage form |
|  | Severe active infections, including untreated or inadequately treated tuberculosis, fungal infections, and varicella-zoster infections |

Abbreviations: BCT, baricitinib; TCZ, tocilizumab; HIV, human immunodeficiency virus; GFR, glomerular filtration rate

**Supplementary Table 2**. Proportions of missing values across the 38 input variables in the development cohort.

| **Features** | **Missing rate (%)** | **Used in Model** |
| --- | --- | --- |
| Urine creatinine | 84.4 |  |
| KL-6 | 52.3 |  |
| Procalcitonin | 35.5 |  |
| Erythrocyte sedimentation rate | 35.4 |  |
| Glucose | 20.3 |  |
| Activated partial thromboplastin time | 15.8 |  |
| Prothrombin time | 12.7 |  |
| Lactic acid | 10.4 |  |
| Partial pressure of oxygen | 9.6 |  |
| Partial pressure of caron dioxide | 9.6 |  |
| Bicarbonate | 9.6 |  |
| pH | 9.6 |  |
| Monocyte | 8.6 |  |
| Neutrophil | 8.6 |  |
| Albumin | 5.7 | TCZ, BCT |
| Alanine aminotransferase | 5.5 |  |
| Aspartate aminotransferase | 5.5 |  |
| WBC (leukocyte) | 4.9 |  |
| Hemoglobin | 4.9 | TCZ, BCT |
| Platelet | 4.9 | TCZ |
| Serum creatinine | 4.9 | TCZ, BCT |
| Height | 4.7 |  |
| Potassium | 4.7 | BCT |
| Blood urea nitrogen | 4.7 | TCZ |
| Sodium | 4.7 |  |
| C-reactive protein | 4.3 |  |
| Blood type | 2.5 |  |
| BMI | 1.8 |  |
| Weight | 1.8 | TCZ, BCT |
| Pulse oxygen saturation | 0.8 |  |
| Body temperature | 0.4 |  |
| Respiratory rate | 0.4 | BCT |
| Age | 0.0 | TCZ, BCT |
| Sex | 0.0 |  |
| Heart rate | 0.0 | BCT |
| Systolic blood pressures | 0.0 |  |
| Diastolic blood pressures | 0.0 | TCZ |
| Pulse pressure | 0.0 |  |

Abbreviations: BCT, baricitinib; TCZ, tocilizumab; KL-6, Krebs von den Lungen-6

**Supplementary Table 3.** Performance of machine learning models predicting treatment outcome of the severe COVID-19 with respect to imputation of missing values.

| Model | Cohort | Imputation Method | ROC-AUC | F1-Score | Accuracy | Sensitivity | Specificity |
| --- | --- | --- | --- | --- | --- | --- | --- |
| TCZ Model | Development | Median | 0.81 | 0.47 | 0.67 | 0.50 | 0.74 |
| TCZ Model | Development | Minimum | 0.77 | 0.41 | 0.65 | 0.43 | 0.74 |
| TCZ Model | Development | Maximum | 0.77 | 0.43 | 0.56 | 0.57 | 0.56 |
| TCZ Model | Development | kNN | 0.77 | 0.42 | 0.60 | 0.5 | 0.65 |
| TCZ Model | Development | MICE | 0.78 | 0.45 | 0.65 | 0.5 | 0.71 |
| TCZ Model | External Test | Median | 0.78 | 0.71 | 0.75 | 1.0 | 0.63 |
| TCZ Model | External Test | Minimum | 0.76 | 0.71 | 0.75 | 1.0 | 0.64 |
| TCZ Model | External Test | Maximum | 0.76 | 0.67 | 0.69 | 1.0 | 0.55 |
| TCZ Model | External Test | kNN | 0.76 | 0.71 | 0.75 | 1.0 | 0.64 |
| TCZ Model | External Test | MICE | 0.71 | 0.77 | 0.75 | 1.0 | 0.57 |
| BCT Model | Development | Median | 0.84 | 0.67 | 0.77 | 1.0 | 0.71 |
| BCT Model | Development | Minimum | 0.80 | 0.67 | 0.77 | 1.0 | 0.71 |
| BCT Model | Development | Maximum | 0.80 | 0.64 | 0.74 | 1.0 | 0.67 |
| BCT Model | Development | kNN | 0.79 | 0.60 | 0.74 | 0.86 | 0.71 |
| BCT Model | Development | MICE | 0.78 | 0.60 | 0.74 | 0.86 | 0.64 |
| BCT Model | External Test | Median | 0.69 | 0.31 | 0.78 | 0.28 | 0.89 |
| BCT Model | External Test | Minimum | 0.70 | 0.32 | 0.79 | 0.28 | 0.9 |
| BCT Model | External Test | Maximum | 0.70 | 0.30 | 0.78 | 0.28 | 0.88 |
| BCT Model | External Test | kNN | 0.68 | 0.32 | 0.79 | 0.28 | 0.9 |
| BCT Model | External Test | MICE | 0.69 | 0.33 | 0.8 | 0.28 | 0.91 |

Abbreviations: BCT, baricitinib; TCZ, tocilizumab; kNN, k-nearest neighbor; MICE, Multiple Imputation by Chained Equations; ROC-AUC, receiver-operating characteristic area under the curve.

**Supplementary Methods**

**Clinical covariates**

Underlying disease data comprised diabetes mellitus, hypertension, cardiovascular disease, chronic kidney, liver, and lung diseases, rheumatic conditions, pregnancy, immunodeficiencies (e.g., HIV infection), solid or hematologic malignancies, and solid organ transplantation. Clinical characteristics encompassed demographic information, medication history, vital signs, National Early Warning Score (NEWS), and laboratory test results. Demographic data included age, sex, height, weight, and body mass index (BMI) recorded at admission. Vital sign data included systolic and diastolic blood pressures, pulse pressure, heart rate, respiratory rate, body temperature, and pulse oxygen saturation, with averages taken from one day prior to the initiation of immunomodulators to the day of initiation. Laboratory test results were similarly derived from average values reported from one day prior to the initiation of immunomodulators to the day of initiation, and included leukocyte, lymphocyte, neutrophil, and platelet counts; hemoglobin; blood urea nitrogen; serum creatinine; aspartate/alanine aminotransferase; albumin; glucose; potassium; sodium; bicarbonate; C-reactive protein; erythrocyte sedimentation rate; procalcitonin; prothrombin time/activated partial thromboplastin time; lactic acid; and arterial blood gas parameters such as pH, partial pressure of oxygen, and partial pressure of carbon dioxide.

**Optimization of ML models**

Patients from the development cohort receiving TCZ (236 cases) or BCT (154 cases), were grouped into training and validation datasets via 5-fold cross-validation using the StratifiedKFold method, to ensure balanced class distributions across folds [21]. Model parameters included *n_estimators* = 200 and *early_stopping_rounds* = 20, optimized using the log loss function. Random states, selected as random integers between 0 and 10,000, were tested to identify the best-performing models in both the training and validation sets. Predictive performance was evaluated by the F1-score during internal validation in the development cohort.
